# A constraint solving approach to model reduction by tropical equilibration

**DOI:** 10.1186/s13015-014-0024-2

**Published:** 2014-12-04

**Authors:** Sylvain Soliman, François Fages, Ovidiu Radulescu

**Affiliations:** Inria, Domaine de Voluceau, Rocquencourt, 78150 France; University of Montpellier 2, Place Eugene Bataillon, Montpellier, 34095 France

**Keywords:** Systems biology, Model reduction, Tropical algebra, Tropical equilibration, Constraint programming

## Abstract

Model reduction is a central topic in systems biology and dynamical systems theory, for reducing the complexity of detailed models, finding important parameters, and developing multi-scale models for instance. While singular perturbation theory is a standard mathematical tool to analyze the different time scales of a dynamical system and decompose the system accordingly, tropical methods provide a simple algebraic framework to perform these analyses systematically in polynomial systems. The crux of these methods is in the computation of tropical equilibrations. In this paper we show that constraint-based methods, using reified constraints for expressing the equilibration conditions, make it possible to numerically solve non-linear tropical equilibration problems, out of reach of standard computation methods. We illustrate this approach first with the detailed reduction of a simple biochemical mechanism, the Michaelis-Menten enzymatic reaction model, and second, with large-scale performance figures obtained on the http://biomodels.net repository.

## Background

Model reduction is a central topic in systems biology and dynamical systems theory, for reducing the complexity of detailed models, finding important parameters, and developing multi-scale models for instance.

Indeed, for many of the problems in computation and analysis of complex systems, the upper limit on the size of the system that can be studied has been reached. This limit can be very low, namely tens of variables for system identification, symbolic calculation or bifurcation of attractors of large dynamical systems. For instance, the complexity of extant symbolic solvers of polynomial equations is exponential in the number of indeterminates and parameters, that sets a drastic limitation to the size of the models that can be analyzed [1,2]. Some examples of computational difficulties that arise when trying to apply standard tools of algebraic geometry to systems biology models can be found in [3]. Model reduction is a way to bypass these limitations by replacing large scale models with models containing less parameters and variables, easier to analyse.

There are mathematical methods, based on singular perturbations or on the theory of invariant manifolds, allowing reduction of fully parametrized systems with separation of time scales. More precisely, in dissipative systems, fast variables relax rapidly to some low dimensional attractive manifold called invariant manifold [4] that carries the slow mode dynamics. A projection of dynamical equations onto this manifold provides the reduced dynamics. Numerical reduction methods, such as computational singular perturbation (CSP, [5]), intrinsic low dimensional manifold (ILDM, [6]) exploit the separation of timescales of various processes and compute approximations of the invariant manifold. Purely structural reduction methods can handle big models possibly with lack of kinetic information [7]. However, the case of biochemical models of intermediate size, with partially known parameters and that ask for symbolic analysis, is more open [8].

While singular perturbation theory is a standard mathematical tool to analyze the different time scales of a dynamical system and decompose the system accordingly, tropical methods provide a simple algebraic framework to perform these analyses systematically in polynomial systems, and in situations when model parameters are known only by their orders of magnitude. Differential equations describing kinetics of biochemical reactions are polynomial or become polynomial after decomposition of reaction mechanisms into elementary steps. For these models, quasi-equilibrium or quasi-steady state invariant manifolds allowing reductions are given by systems of algebraic equations [3]. A potentially crucial application of tropical mathematics is to enumerate and describe asymptotic solutions of algebraic systems of equations [9]. In particular, tropical solutions of polynomial equations provide the leading terms of their solutions via curves or in other terms via Newton-Puiseux series [10,11]. At the basis of our method lies the idea that equilibration of fast variables on invariant manifolds implies, at lowest order, equilibration of at least two dominant monomials, one positive and the other negative in the right hand side of the differential equations. We have called such a condition, similar to Kapranov’s condition [11] for existence of Newton-Puiseux series with specified lowest order terms, tropical equilibration. The crux of our method lies in the computation of tropical equilibrations that define some reduced truncated systems with fewer parameters to identify, thus pointing to fewer experiments to (in)validate the model [12,13]. Our method copes with uncertain parameters by replacing exact values by orders of magnitude and the reduction is performed symbolically in both variables and parameters. With respect to methods based on singular perturbations, this could be less precise at lowest order, but it is more general in implementation.

Solving the tropical equilibration problem boils down to solving a system of equations in the min-plus algebra (also known as the tropical semiring). For solving linear tropical systems there are pseudo-polynomial algorithms, i.e. whose complexity is polynomial in the size of the system and in the absolute values of its coefficients [14]. In the nonlinear case, the existence of tropical equilibrations, which is equivalent to the problem of the intersection of tropical varieties, was shown to be NP-complete [15]. In this paper we show that constraint-based methods, using reified constraints for expressing the equilibration conditions, make it possible to numerically solve non-linear tropical equilibration problems, out of reach of standard computation methods.

We first illustrate this approach with the detailed reduction of a simple biochemical mechanism, the Michaelis-Menten enzymatic reaction model. We detail the general procedure to obtain truncated systems by identification, through equilibration, of fast and slow species, and relate the obtained reduced systems to the usual notions of quasi-steady-state and quasi-equilibrium. Then, we demonstrate that the approach is computationally feasible, and scales up properly, by treating in an automatic way all the curated dynamical models of http://biomodels.net repository [16].

## Model reduction by tropicalization

We consider networks of biochemical reactions with mass action kinetic laws. The structure of a reaction is defined by a multiset rewriting rule as

$$\sum_{i=1}^{n} \alpha_{ji} A_{i} \rightarrow \sum_{k=1}^{n} \beta_{jk} A_{k} $$

where *A*_*i*_, *i*=1,…,*n* denote the chemical species and *α*_*ji*_, *β*_*jk*_ are positive integers named stoichiometric coefficients defining which species are consumed and produced by the reaction *j*, 1≤*j*≤*r*, and in which quantities.

The mass action law means that reaction rates are monomial functions of the species concentrations *x*_*i*_, 1≤*i*≤*n* and read

$$ R_{j}(\boldsymbol{x}) = k_{j} \boldsymbol{x}^{\boldsymbol{\alpha}_{j}}, $$

where *k*_*j*_>0 are kinetic parameters and we use the shorthand notation $\boldsymbol {x}^{\boldsymbol {\alpha }_{j}} = x_{1}^{\alpha _{j1}} \ldots x_{n}^{\alpha _{\textit {jn}}}$.

The network dynamics is then described by the following differential equations

(1) $$ \frac{{dx}_{i}}{dt} = \sum_{j=1}^{r} k_{j} S_{ij} \boldsymbol{x}^{\boldsymbol{\alpha_{j}}}.   $$

where *S*_*ij*_=*β*_*ji*_−*α*_*ji*_ are entries of the stoichiometric matrix.

In what follows, the kinetic parameters do not have to be known precisely, they are given by their orders of magnitude. A convenient way to represent orders is by considering that

$$k_{j} = \bar k_{j} \epsilon^{\gamma_{j}}, $$

where *ε* is a positive parameter much smaller than 1, *γ*_*j*_ is an integer or, more generally, a rational number, and $\bar k_{j}$ has order unity. An approximate integer order can be obtained from any real positive parameter by

$$\gamma_{j} = \text{round}(\log(k_{j}) / \log(\epsilon)), $$

where round stands for the closest integer. Notice that in this representation, small quantities have large orders. Furthermore, the smaller *ε*, the better the separation between quantities of different orders, indeed ${\lim }_{\epsilon \to 0} \frac {k_{i}}{k_{j}} = \infty $, if *γ*_*i*_<*γ*_*j*_.

We also define orders for species concentrations, using a vector of orders ***a***=(*a*_1_,…,*a*_*n*_), such that $\boldsymbol {x} = \bar {\boldsymbol {x}} \epsilon ^{\boldsymbol {a}}$. We suppose that various (*a*_1_,…,*a*_*n*_) are integers or rational numbers with a common denominator. In our method we will calculate the concentration orders as solutions of the tropical equilibration problem (see below).

First, let us replace Eqs. (1) by their equivalent rescaled versions

(2) $$ \frac{d\bar{x}_{i}}{dt} = \sum\limits_{j=1}^{r} \epsilon^{\mu_{j} - a_{i}} k_{j} S_{ij} {\bar{\boldsymbol{x}}}^{\boldsymbol{\alpha_{j}}},   $$

where

(3) $$ \mu_{j} = \gamma_{j} + <\boldsymbol{a},\boldsymbol{\alpha_{j}}>,   $$

and <,> stands for the vector dot product.

The r.h.s. of each equation in (2) is a sum of multivariate monomials in the concentrations. The orders *μ*_*j*_ indicate how large are these monomials, in absolute value. For sufficiently small *ε*, monomials of different orders are well separated. For instance a monomial of smallest order $\mu _{j} < \mu _{j^{\prime }}$ dominates the other monomials $k_{j} |S_{\textit {ij}}| \boldsymbol {x}^{\boldsymbol {\alpha _{j}}} \gg k_{j^{\prime }} |S_{ij^{\prime }}| \boldsymbol {x}^{\boldsymbol {\alpha _{j'}}}$. One could see all these monomials as “forces” acting on the chemical species. At steady state, the resultant of all these forces should be naught. A consequence of this is that the orders of dominant positive and negative forces should be equal. This is exactly our notion of *tropical equilibration* that we introduced in [13]. More precisely, we say the system (2) is *tropically equilibrated* iff

(4) $$ \min(\mu_{j}, S_{ij} < 0) = \min(\mu_{j'}, S_{ij'} > 0),\, \text{for all} \quad i = 1,\ldots,n   $$

The tropical equilibration problem consists in solving the system (4) for orders *a*_*i*_, 1≤*i*≤*n*.

Another way to understand the condition (4) is via Newton-Puiseux series. Suppose we want to solve the polynomial equation

(5) $$ P(x,\epsilon) = \sum_{j} b_{j} \epsilon^{\gamma_{j}} x^{\alpha_{j}} =0,   $$

where *α*_*j*_ are positive integers, *γ*_*j*_ are rational powers, and *b*_*j*_ are real coefficients. It is well known [10] that solutions of this equation can be expressed as Newton-Puiseux series, i.e. have the form

$$x(\epsilon) = c_{1} \epsilon^{\frac{a_{1}}{q}} + c_{2} \epsilon^{\frac{a_{2}}{q}} +\ldots, $$

where *c*_*i*_ are complex coefficients, *a*_1_<*a*_2_<… are integers, *q* is a positive integer. By substituting $x(\epsilon) = c_{1} \epsilon ^{\frac {a_{1}}{q}}(1 + x_{1}(\epsilon))$ (where *x*_1_(*ε*) collects terms with positive orders in *ε*) in (5) we get

$$P(x,\epsilon) = \sum_{j} b_{j} c_{1}^{\alpha_{j}} \epsilon^{\gamma_{j} + a_{1} \alpha_{j}/q} + r_{1}(\epsilon) =0, $$

where *r*_1_(*ε*) collects higher order terms. Necessary conditions for *P*(*x*,*ε*)=0 read at lowest order

(6) $$ \sum_{j,\gamma_{j} + a_{1} \alpha_{j}/q = m} b_{j} c_{1}^{\alpha_{j}} = 0   $$

(7) $$ m = \min_{j}(\gamma_{j} + a_{1} \alpha_{j}/q)   $$

In order to satisfy (6), the minimum in (7) should be attained at least twice. Furthermore, if one looks for real solutions $c_{i} \in \mathbb {R}$, then from (6) it follows that at least two *b*_*j*_ corresponding to the minimum (7) should have opposite signs. This means that the lowest order *a*_1_/*q* in the Newton-Puiseux series solution has to satisfy a tropical equilibration problem.

We must emphasize that the tropical equilibration condition is weaker than the steady state condition, and makes sense also away from a steady state. In systems with slow/fast variables, the fast variables are equilibrated by compensation of dominant forces whose orders result from the tropical condition (4). As a consequence, the fast variables can be expressed as functions of the slow variables. However, both fast and slow variables can slowly evolve under the influence of weaker, higher order forces. This picture is valid as long as the relative dominance relations between various monomial terms in Eqs.(2) are preserved. This is true if the rescaled concentrations $\bar {x}_{i}$ stay between bounds, whereas *ε* is allowed to tend to zero.

To summarize, the tropical equilibration is a necessary condition for the elimination of fast variables and model reduction. As we showed in [13], in order to become sufficient this condition should be combined with a boundedness condition, called permanency:

### **Definition0.1**.

The system (1) is permanent, if there are two constants *C*_−_>0 and *C*_+_>0, a set of renormalization exponents *a*_*i*_, and a function *T*_0_ of the initial conditions, such that after renormalization we have

$$ C_{-} < \bar x_{i}(t) < C_{+}, \quad for \ all \ t > T_{0} (\boldsymbol{x}(0)) \ and \ for \ every \ i. $$

## A simple example, the Michaelis-Menten reduction

The Michaelis-Menten enzymatic reaction network consists of three reactions:

$$S + E \underset{k_{-1}}{ \overset{k_{1}}{\rightleftharpoons}} ES \overset{k_{2}}{\rightarrow} P + E, $$

where *S*,*ES*,*E*,*P* represent the substrate, the enzyme-substrate complex, the enzyme and the product, respectively.

The corresponding system of polynomial differential equations reads:

(8) $$ \begin{aligned} x_{1}^{\prime} & = -k_{1} x_{1} x_{3} + k_{-1} x_{2} \\ x_{2}^{\prime} & = k_{1} x_{1} x_{3} - (k_{-1}+k_{2})x_{2} \\ x_{3}^{\prime} & = -k_{1} x_{1} x_{3} + (k_{-1} + k_{2}) x_{2} \\ x_{4}^{\prime} & = k_{2} x_{2} \end{aligned}  $$

where *x*_1_= [ *S*], *x*_2_= [ *ES*], *x*_3_= [ *E*], *x*_4_= [ *P*].

It can be easily checked that the system has two algebraic invariants: (*x*_2_+*x*_3_)^′^=0, which implies

(9) $$ x_{2} + x_{3} = e_{0},   $$

where *e*_0_ is a positive constant (the total amount of enzyme), and

(10) $$ x_{1}+x_{3}+x_{4}= s_{0}   $$

where *s*_0_ is a positive constant (the total amount of substrate and product). These conservation laws can be used to reduce the model by elimination of *x*_4_ (by (10)) and *x*_3_ (by (9)). It follows:

(11) $$ \begin{aligned}  x_{1}^{\prime} & = -k_{1} x_{1}(e_{0}-x_{2}) + k_{-1} x_{2} \\ x_{2}^{\prime} & = k_{1} x_{1} (e_{0} - x_{2}) - (k_{-1}+k_{2})x_{2} \end{aligned}  $$

The constraint *x*_2_≤*e*_0_ resulting from the elimination is also to consider, but we will see that all equilibrations of the above equations already imply it.

### Tropical equilibration equations

By rescaling variables and parameters, we get $x_{i}= \bar x_{i} \epsilon ^{a_{i}}$, 1≤*i*≤2, $k_{1}= \bar k_{1} \epsilon ^{\gamma _{1}}$, $k_{-1}= \bar k_{-1} \epsilon ^{\gamma _{-1}}$, $e_{0}= \bar e_{0} \epsilon ^{\gamma _{e}}$.

The tropical equilibration equations for the reduced system read:

(12) $$ \gamma_{1}+ \gamma_{e} + a_{1} = \min (\gamma_{1} + a_{1},\gamma_{-1}) + a_{2}   $$

(13) $$ \gamma_{1}+ \gamma_{e} + a_{1} = \min (\gamma_{1}+ a_{1}, \min(\gamma_{-1},\gamma_{2})) + a_{2}   $$

The set of integer (or rational) orders endowed with the ⊕= min and ⊗=+ operations is a semi-field, called min-plus algebra or tropical semi-field [17]. In this semi-field, −*∞* plays the role of 0 and 0 plays the role of 1. The multiplicative inverse of *a* is denoted −*a*. Our tropical equilibration problem means solving a set of polynomial equations in this semi-field. Using these notations and properties of semi-field operation, the tropical equations become:

(14) $$ \gamma_{1} \otimes \gamma_{e} \otimes a_{1} \otimes (-a_{2}) = (\gamma_{1} \otimes a_{1}) \oplus \gamma_{-1}   $$

(15) $$ \gamma_{1} \otimes \gamma_{e} \otimes a_{1} \otimes (-a_{2}) = (\gamma_{1} \otimes a_{1}) \oplus \gamma_{-1} \oplus \gamma_{2}   $$

### Classical Michaelis-Menten reduction

The classical derivation of the Michaelis-Menten reduction is based on the behaviour of the variable *x*_2_ for the complex concentration. Using (8) it follows that:

$$x_{4}^{\prime} = V_{m} x_{2} / e_{0} $$

where *V*_*m*_=*k*_2_*e*_0_ is the maximum value of the production rate $x_{4}^{\prime }$, since *x*_2_≤*e*_0_.

The variable *x*_2_ satisfies equilibration relations and can be expressed as a function of a slow variable (either the substrate *x*_1_ when *x*_2_ is small, or the sum *x*_1_+*x*_2_ in general) in two situations: quasi-stationarity and quasi-equilibrium.

The quasi-stationarity corresponds to setting $x_{2}^{\prime }$ to zero and is justified by the smallness of *x*_2_ that can be considered a fast species (radical). More precisely one has *k*_1_*x*_1_(*e*_0_−*x*_2_)−(*k*_−1_+*k*_2_)*x*_2_=0, leading to *x*_2_=*x*_1_*e*_0_/(*K*_*m*_+*x*_1_), where *K*_*m*_=(*k*_−1_+*k*_2_)/*k*_1_, i.e.

(16) $$ x_{4}^{\prime} = V_{m} x_{1} / (K_{m}+x_{1})   $$

The quasi-equilibrium corresponds to setting *k*_1_*x*_1_(*e*_0_−*x*_2_)−*k*_−1_*x*_2_ = 0, meaning zero net flux of the first reaction in the mechanism. This leads to *x*_2_ =*x*_1_*e*_0_/((*k*_−1_/*k*_1_)+*x*_1_), i.e.

(17) $$ x_{4}^{\prime} = V_{m} x_{1} / ((k_{-1}/k_{1}) +x_{1})   $$

This is justified by having a very fast transformations in the direct and reverse sense by the first reaction, much faster than the transformations by the second reaction. In this case both *x*_1_ and *x*_2_ are fast, but their sum *x*_1_+*x*_2_ is slow.

We show next, in Section “Tropical equilibrations and model reductions”, that analysis of tropical equations provide the conditions for the asymptotic validity of quasi-stationarity and quasi-equilibrium approximations and also the exhaustive list of asymptotic regimes.

### Geometrical interpretation

**Figure 1:**
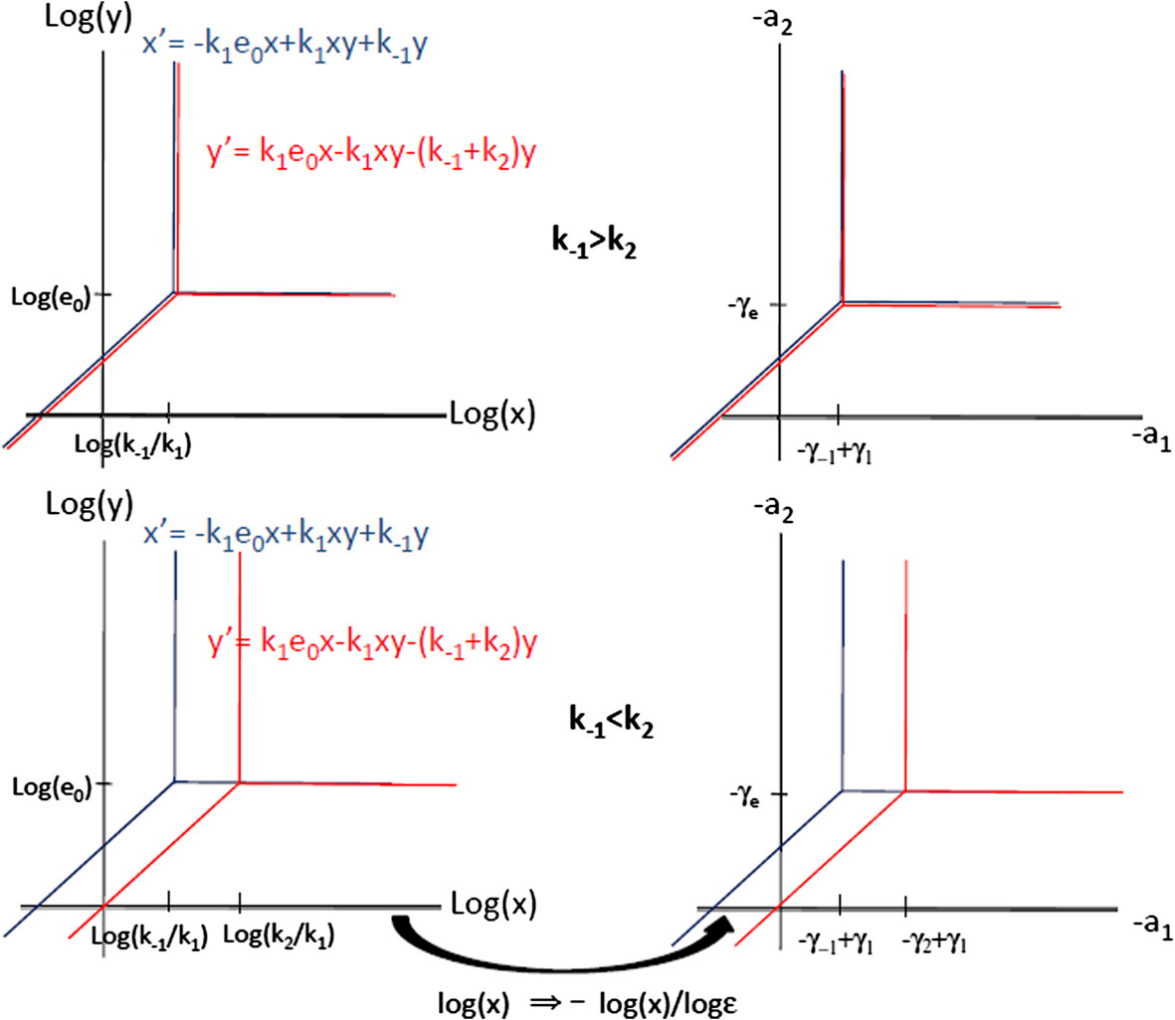
**Tropical curves in the planes of concentrations and orders for the two variables Michaelis-Menten model.** Tropical curves are defined as the locus of points where two monomials of a polynomial describing a differential equation are equal. The tropical curves for each differential equations are indicated by colors, blue for the first equation and red for the second equation. The vertical half-line of each of the tripods does not carry tropical equilibrations because it corresponds to equality of two monomials of the same sign. The horizontal and the oblique half-lines of the tripods carry tropical equilibrations. We have represented the two situations when *k*_−1_>*k*_2_ and when *k*_−1_<*k*_2_. All the tropical equilibrations are double (both variables are equilibrated) in the first case, and can be simple (only one variable is equilibrated) in the latter.

It was discussed in [13] that there is a bijection between the set of solutions of each tropical equation and parts of the tropical curves of the polynomials defining the ordinary differential equations. A tropical curve is defined as the locus of species concentration values (*x*,*y*) where at least two monomials of the considered polynomial are equal and larger than all the others. In logarithmic scale, this locus is made of lines, half-lines, or line segments [13,18]. There is one tropical curve for each differential equation. For instance, the tropical curve defined by the polynomial −*k*_1_*e*_0_*x*_1_+*k*_1_*x*_1_*x*_2_+*k*_−1_*x*_2_ is made of three half-lines with a common origin depicted in Figure 1, namely

(18) $$  \log (x_{2}) = \log(e_{0}),\quad\qquad \log (x_{1}) > \log(k_{-1} / k_{1})  \\  $$

(19) $$ \log (x_{1}) = \log(k_{-1}/k_{1}),\qquad \log (x_{2}) > \log(e_{0})  \\  $$

(20) $$ \log (x_{2}) = \log (x_{1}) + \log(e_{0} k_{1}/k_{-1}), \;\;\log (x_{1}) < \log(k_{-1}/k_{1})   $$

The solutions of the tropical equation (14) form two branches, corresponding to the two situations (*γ*_1_⊗*a*_1_)⊕*γ*_−1_=(*γ*_1_⊗*a*_1_) and (*γ*_1_⊗*a*_1_)⊕*γ*_−1_=*γ*_−1_, respectively. These are two half-lines in the plane of concentration orders:

(21) $$ a_{2} = \gamma_{e},\qquad\qquad\qquad\qquad\qquad\qquad\quad \gamma_{1} + a_{1} < \gamma_{-1}  \\  $$

(22) $$ a_{2} = a_{1}+ \gamma_{1} + \gamma_{e} - \gamma_{-1}, \qquad\qquad\qquad\gamma_{1} + a_{1} > \gamma_{-1}   $$

The two branches of solutions can be also related to parts of the tropical curves corresponding to the equilibration of monomials of different signs. More precisely (21) corresponds to (18), and (22) corresponds to (20). The branch (19) of the tropical curve corresponds to the equality of two positive monomials and has no correspondence in the set of tropical equilibrations.

Similarly to computing steady states as intersections of null-clines, we are considering multiple tropical equilibrations as intersections of tropical curves.

We therefore consider the second tropical equation (15), in two situations *γ*_−1_⊕*γ*_2_=*γ*_−1_ and *γ*_−1_⊕*γ*_2_=*γ*_2_. In the first case the tropical equation (15) is equivalent to the tropical equation (14) (also, the tropical curves coincide). Therefore, the two solutions (21) and (22) equilibrate both equations. In the second case, the solutions of the tropical equation (15) form two branches, corresponding to (*γ*_1_⊗*a*_1_)⊕*γ*_2_=*γ*_1_⊗*a*_1_ and (*γ*_1_⊗*a*_1_)⊕*γ*_2_=*γ*_2_, respectively. They correspond to two half-lines in the plane of orders (*a*_1_,*a*_2_), namely *a*_2_=*γ*_*e*_, *a*_1_<*γ*_2_−*γ*_1_ and *a*_2_=*a*_1_+*γ*_1_+*γ*_*e*_−*γ*_2_, *a*_1_>*γ*_2_−*γ*_1_. A simple graphical inspection of the relative positions of these half-lines with respect to the half-lines carrying solutions of the first tropical equation shows that there are four branches of tropical equilibrations:

(23) $$ a_{2} = \gamma_{e}, \qquad\qquad a_{1} < \gamma_{2} - \gamma_{1}  $$

(24) $$ a_{2} = \gamma_{e},\qquad\qquad \gamma_{2} - \gamma_{1} < a_{1} < \gamma_{2} - \gamma_{1}  $$

(25) $$ a_{2} = a_{1}+ \gamma_{1}+ \gamma_{e} - \gamma_{2},\qquad\qquad\!\! a_{1} > \gamma_{2} - \gamma_{1}  $$

(26) $$ a_{2} = a_{1}+ \gamma_{1}+ \gamma_{e} - \gamma_{-1},\qquad\qquad a_{1} > \gamma_{-1} - \gamma_{1}  $$

The branch (23) equilibrates the two variables. The branch (25) equilibrates only the second variable, whereas the branches (24), (26) equilibrate only the first variable.

### Tropical equilibrations and conservation laws

The reduced Michaelis-Menten mechanism with two dynamical variables has been obtained by elimination of a variable using an exact conservation law. It is interesting to compute the tropical equilibrations directly, in the unreduced model. In this three variables model, two of the equilibrium equations are identical. Like for computation of steady states, we need the conservation law as an extra constraint. If we treat this constraint exactly, we obtain the reduced model. An approximate treatment of Eqs. (8), (9), considering equilibration of dominant terms, leads to the tropical problem:

(27) $$ \gamma_{1} \otimes a_{1} \otimes a_{3} = \gamma_{-1} \otimes a_{2}   $$

(28) $$ \gamma_{1} \otimes a_{1} \otimes a_{3} = (\gamma_{-1} \otimes a_{2})\oplus (\gamma_{2} \otimes a_{2})   $$

(29) $$ a_{2} \oplus a_{3} = \gamma_{e}   $$

This tropical problem is different from (14), (15) and leads to different solutions in general. Firstly, let us notice that elimination is not possible in semi-fields, because there is no additive inverse in general. Hence, (27), (28) (29) can not be reduced to an equivalent system of two tropical equations. Secondly, dominant monomial equilibration in the reduced model does not always correspond to monomial equilibrations in the unreduced model. A typical example is the monomial *x*_1_*x*_3_ that becomes the difference *x*_1_*e*_0_−*x*_1_*x*_2_ in the reduced model. The two monomials can equilibrate each other in the reduced model, but the same quantity is an unique, un-equilibrated monomial in the full model.

There are six branches of tropical solutions of the system (27), (28), (29). Two branches are obtained when *γ*_−1_⊗*a*_2_=*γ*_−1_. In this case the two tropical equations (27), (28) are identical. Depending on *a*_2_⊕*a*_3_=*a*_2_, or *a*_2_⊕*a*_3_=*a*_3_ we get:

(30) $$ a_{2} = \gamma_{e},\qquad\quad a_{1} < \gamma_{-1} - \gamma_{1}   $$

(31) $$ a_{2} = a_{1}+ \gamma_{1}+ \gamma_{e} - \gamma_{-1},\qquad\qquad\qquad a_{1} > \gamma_{-1} - \gamma_{1}   $$

These branches correspond to equilibrations of all the variables.

When *γ*_−1_⊗*a*_2_=*γ*_2_ the two tropical Eqs. 27, (28) are incompatible. Depending on *a*_2_⊕*a*_3_=*a*_2_, or *a*_2_⊕*a*_3_=*a*_3_ and further choosing only one of the two tropical Eqs. 27, (28) we get the following branches:

(32) $$ a_{2} = \gamma_{e},\qquad\qquad a_{1} < \gamma_{-1} - \gamma_{1}   $$

(33) $$ a_{2} = \gamma_{e},\qquad\qquad a_{1} < \gamma_{2} - \gamma_{1}   $$

(34) $$ a_{2} = a_{1}+ \gamma_{1}+ \gamma_{e} - \gamma_{-1},\qquad\qquad a_{1} > \gamma_{-1} - \gamma_{1},   $$

(35) $$ a_{2} = a_{1}+ \gamma_{1}+ \gamma_{e} - \gamma_{2},\qquad\qquad a_{1} > \gamma_{2} - \gamma_{1}   $$

In the branches (32), (34), the variables *x*_2_, *x*_3_ are not equilibrated, whereas in the branches (33), (35), the variable *x*_1_ is not equilibrated.

Comparison of Eqs. (30)-(35) and Eqs. (21)-(26) proves that the tropical equations of the unreduced model have the same set of solutions as the reduced model. However, the branch of solutions (33) equilibrates all the variables in the reduced model and does not equilibrate the variable *x*_1_ in the reduced model. The reason is exactly the one given above: the monomial *x*_1_*x*_3_ is dominant and un-equilibrated in the unreduced model, becomes *x*_1_*e*_0_−*x*_1_*x*_2_ with equilibrated monomials in the reduced model.

### Tropical equilibrations and model reductions

Tropical equilibrations can be used for model reduction. The reduction starts by tropical truncation. We call tropically truncated model the model obtained by elimination of all dominated monomials from the r.h.s. of the ordinary differential equations. The next step is ordering the variables according to the values of the exponents *μ*_*i*_−*a*_*i*_. This allows to determine which variables are slow and fast.

An additional construction is needed in the case when the tropically truncated system of fast variables has conservation laws that are not conserved by the un-truncated system. The conservation laws define species pools that are supplementary slow variables. The pools follow differential equations involving previously dominated monomials.

For instance, in the two variables Michaelis-Menten model, we found essentially two types of reduced models, corresponding to quasi-equilibrium and quasi-stationarity approximations [19].

The branch (21) of tropical solutions leads to the following truncated system:

(36) $$ \begin{aligned} x_{1}^{\prime} & = - k_{1} x_{1} e_{0} + k_{-1} x_{2}\\ x_{2}^{\prime} & = k_{1} x_{1} e_{0} - k_{-1} x_{2} \end{aligned}  $$

This truncated system has conserved quantity *z*=*x*_1_+*x*_2_. The variable *z* is not conserved by the full model described by (11). Indeed, addition of Eqs. (11) leads to *z*^′^=−*k*_−1_*x*_2_. As the variable *x*_2_ can be eliminated from −*k*_1_*x*_1_*e*_0_+*k*_−1_*x*_2_=0 and *x*_1_+*x*_2_=*z* we have the reduced dynamics *z*^′^=−*k*_*z*_*z*, where *k*_*z*_=*k*_−1_/(1+*k*_−1_/(*k*_1_*e*_0_)). For small *ε*, we can consider that $\phantom {\dot {i}\!}k_{z} \sim \epsilon ^{\gamma _{z}}$, with *γ*_*z*_=*γ*_−1_− min(0,*γ*_−1_−*γ*_1_−*γ*_*e*_). Because *μ*_1_−*a*_1_=*γ*_1_+*γ*_*e*_, *μ*_2_−*a*_2_=*γ*_1_+*γ*_*e*_+*a*_1_−*a*_2_=*γ*_−1_ the relation *k*_*z*_>*μ*_1_−*a*_1_, *k*_*z*_>*μ*_2_−*a*_2_ are always satisfied guaranteeing that *z* is slower than *x*_1_, *x*_2_. The form (36) of the truncated equations and the conservation of *x*_1_+*x*_2_ by the fast dynamics shows that this case corresponds to quasi-equilibrium of the first reaction in the Michaelis-Menten model, as described in Section “Classical Michaelis-Menten reduction”, equation 17.

The branches (23) and (24) lead to quasi-equilibrium with the following truncated system:

(37) $$ \begin{aligned} x_{1}^{\prime} & = - k_{1} x_{1} (e_{0} - x_{2})\\ x_{2}^{\prime} & = k_{1} x_{1} (e_{0} - x_{2}) \end{aligned}  $$

These cases correspond to saturation of the enzyme (*x*_2_ has the same order as *e*_0_). A slow variable *z*=*x*_1_+*x*_2_ has to be introduced as before, but the reduced dynamics is *z*^′^=−*k*_−1_*x*_2_=−*k*_−1_*e*_0_.

The branch (25) leads to quasi-stationarity of the enzyme/substrate complex. In this case we have the tropical truncated system:

(38) $$ \begin{aligned} x_{1}^{\prime} & = - k_{1} x_{1} e_{0} \\ x_{2}^{\prime} & = k_{1} x_{1} e_{0} - k_{2} x_{2} \end{aligned}  $$

The variable *x*_1_ is not equilibrated, which still allows for model reduction because this variable is slow. The fast variable *x*_2_ is equilibrated, and the equilibration equation corresponds to the classical notion of quasi-stationary approximation, as described in Section “Classical Michaelis-Menten reduction”, equation 16. In this case, *μ*_1_−*a*_1_=*γ*_1_+*γ*_*e*_, *μ*_2_−*a*_2_=*γ*_1_+*γ*_*e*_+*a*_1_−*a*_2_=*γ*_2_. The condition that *x*_1_ is slower than *x*_2_ reads *μ*_1_−*a*_1_>*μ*_2_−*a*_2_ and we get the additional condition *γ*_1_+*γ*_*e*_>*γ*_2_, which is a low enzyme concentration condition.

The branch (26) leads to quasi-stationarity of the substrate with the following truncated system:

(39) $$ \begin{aligned} x_{1}^{\prime} & = - k_{1} x_{1} e_{0} + k_{-1} x_{2},\\ x_{2}^{\prime} & = - k_{2} x_{2} \end{aligned}  $$

The variable *x*_2_ is not equilibrated, which is allowed only if this variable is slower than *x*_1_. In this case, *μ*_1_−*a*_1_=*γ*_1_+*γ*_*e*_, *μ*_2_−*a*_2_=*γ*_2_. The condition that *x*_2_ is slower than *x*_1_ reads *μ*_1_−*a*_1_<*μ*_2_−*a*_2_ and leads to the additional condition *γ*_1_+*γ*_*e*_<*γ*_2_, which is a high enzyme concentration condition.

Finally, the branch (24) leads to the truncated system:

(40) $$ \begin{aligned} x_{1}^{\prime} & = - k_{1} x_{1} (e_{0} - x_{2}) \\ x_{2}^{\prime} & = - k_{2} x_{2} \end{aligned}  $$

The variable *x*_1_ is equilibrated, but it can not satisfy permanency. Indeed, at fixed *x*_2_ this variable will converge to zero. Therefore, this tropical equilibration does not lead to a reduced model.

## Tropical equilibration as a constraint satisfaction problem

As explained in Section “Model reduction by tropicalization”, given a biochemical reaction system with its Mass-Action kinetics, and a small *ε*, the problem of tropical equilibration is to look for a rescaling of the variables such that the dominating positive and negative term in each ODE *equilibrate* as per Eqs. (4), i.e., are of the same degree in *ε*.

Section “A simple example, the Michaelis-Menten reduction” showed that it is possible to iteratively reduce the equilibration problem to a linear system of equations for each possible pair of positive and negative dominating monomial. It is actually possible to consider fewer pairs by restricting that search to the pairs denoting edges of the Newton polygon [13]. Nevertheless, the number of linear systems to consider remains exponential in the number of species, and may lead to redhibitory computational costs, especially when handling biochemical systems with hub molecules, i.e., molecules involved in a high number of reactions (e.g., E2F, p53, cMyc in cell-cycle control or NF *κ*B in signalling), which corresponds to a Newton polygon with many vertices.

In order to tackle that complexity, we propose a numerical approach based on Constraint Programming, that encodes the equilibration problem as a Constraint Satisfaction Problem (CSP) [20-22] and uses reified constraints to prune the search space. Constraint Programming is a paradigm that has showed great success at solving combinatorial decision or optimization problems, in particular for real-world instances of NP-hard problems, e.g., in the field of production planning and scheduling. It is therefore an interesting way to approach the combinatorial explosion described above.

In presence of invariants (conservation laws) in the original system, Section “Conservation law constraints” has shown that some constraints related to rescaling need be added. We have shown in [23] that finding these conservation laws can be efficiently solved by constraint methods. Here we will thus assume that the conservation laws are given in input. In our prototype implementation, both the computation of conservation laws and the following equilibration are performed for a given system.

### Reified constraints

Key to the modeling of tropical equilibration problems as CSP are reified constraints. Reified constraints are special constraints that link in a bidirectional way the value of a boolean variable to the satisfaction of another constraint. They allow for powerful cuts in the search space by propagating the truth value of some constraints of the problem to the truth value of the Boolean variable, and vice versa.

For instance, the reified constraint

$$\mathrm{B} \#<==> \mathrm{X}\#=\mathrm{Y} $$

states that the Boolean variable *B* is true (i.e. equal to 1) if and only if the constraints *X*=*Y* is satisfied. That constraint posts the constraint *X*=*Y* (resp. *X*≠*Y*) as soon as *B* gets value 1 (resp. 0), and vice versa, sets *B*=1 (resp. *B*=0) as soon as *X*=*Y* (resp. *X*≠*Y* i.e. when the domains of *X* and *Y* become disjoint).

For the tropical equilibration problem, these constraints are at the core of our representation of the minimum constraints as they enforce the propagation of existing knowledge before branching on the two possible values. Indeed, if *A* is the minimum of *B* and *C*, you can derive many things on the domains of *A*, *B* and *C* before eventually trying *A*=*B* or *A*=*C*. For instance it is safe to add that *A*≤*B* and *A*≤*C*, but also if you have, from other equations, that *B*≥*min*_*B*_ and *C*≥*min*_*C*_ then you can add the fact that *A*≥*min*(*min*_*B*_,*min*_*C*_). If later you obtain that actually *A*=*B* then you can enforce *C*≥*B*, etc. Section “Minimum constraints” shows in more detail how reified constraint do precisely this kind of conditional addition of cuts and can therefore be used to handle minimum constraints while postponing enumerative search as much as possible.

### Variables and domains

**Figure 2:**
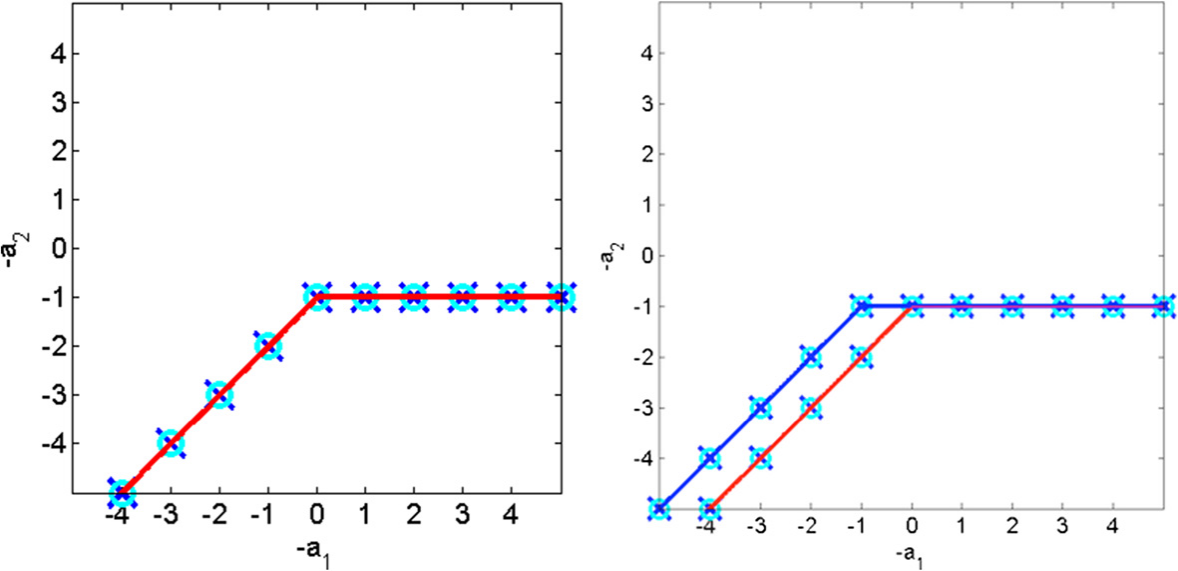
**Comparison of the theoretical and computed equilibrations in the cases** ***k***_**−1**_ **>** ***k***_**2**_ **and** ***k***_**−1**_ **<** ***k***_**2**_ **.** The circles are equilibrations computed for the simplified two variables Michaelis-Menten model, the crosses are for the full three variables model. The lines indicate the theoretical equilibrations.

For practical reasons, mainly the lack of an efficient solver over rationals with reified constraints, we use a finite domain solver and therefore only look for integer solutions (whereas solutions are rational). In practice this did not seem to change much the nature of results, see Figure 2. Extensions of the approach to cope with half-integer solutions or with rational solutions with a common, small denominator are straightforward.

For each original equation *dx*_*i*_/*dt*, 1≤*i*≤*n* is introduced a variable $a_{i}\in \mathbb {Z}$ that is used to rescale the system by posing $x_{i} = \epsilon ^{a_{i}}\bar {x_{i}}$. These are the variables of our CSP. Note that they require a solver handling  like for instance SWI-Prolog [24,25] with the clpfd library by Markus Triska, which we used for our implementation.

### Tropical equilibration constraints

For each differential equation that should be equilibrated is a list of positive monomials $M^{+}_{i}$, and a list of negative monomials $M^{-}_{i}$. The degrees in *ε* of all these monomials are integer linear expressions in the *a*_*i*_. Now, to obtain an equilibration one should enforce for each *i* that the minimum degree in $M^{+}_{i}$ is equal to the minimum degree in $M^{-}_{i}$. This corresponds to the Eqs. 4. We will see how they can be implemented with reified constraints, but for now, let us assume a constraint min(L, M)| that enforces that the variable M of  is the minimum value of a list L of linear expressions over variables of . We have in our CSP, for each 1≤*i*≤*n*, min(PositiveMonomialDegrees, M) and min(NegativeMonomialDegrees, M).

### Conservation law constraints

The second kind of constraint comes from conservation laws. Each conservation law is an equality between a linear combination of the *x*_*i*_ and a constant *c*_*i*_. By rescaling, we obtain a sum of rescaled monomials equal to $\epsilon ^{\log (c_{i})/\log (\epsilon)}\bar {c_{i}}$. We want this equality to hold when *ε* goes to zero, which implies that the minimal degree in *ε* in the left hand side is equal to (the round of) the degree of the right hand side. Since once again the degrees on the left are linear combinations of our variables *a*_*i*_, this is again a constraint of the form: min(ConservationLawDegrees, K) where K is equal to round(log(*c*_*i*_)/ log(*ε*)). This corresponds to the tropical equation (29).

### Minimum constraints

Furthermore, if the system under study is not at steady state, the minimum degree should not be reached only once, which would lead to a constant value for the corresponding variable when *ε* goes to zero, but at least twice. This is the case for the example treated in [12]. The constraint we need is therefore slightly more general than min/2: we need the constraint min(L, M, N) which is true if M is smaller than each element of L and equal to N elements of that list. Note that using CLP notation, we have:

$$\text{min}(\mathrm{M}, \mathrm{L})\quad :-\quad \mathrm{C}\#>=1,\quad \text{min}(\mathrm{M}, \mathrm{L}, \mathrm{C}). $$

In order to enforce that the minimum is reached at least a required number of times, one obvious solution is to try all pairs of positive and negative monomials and count the successful pairs [26]. However, this is not necessary, the min(L, M, N) constraint directly expresses the cardinality constraint on the minimums and can be implemented using *reified constraints* to propagate information between L, M and N in all directions, without enumeration. Using SWI-Prolog notations, the implementation of min/3 by reified constraints is as follows:

$$\begin{aligned} &\text{min}([],_, 0).\\ &\text{min}([\mathrm{H} \mathrm{T}], \mathrm{M}, \mathrm{C}) :- \mathrm{M}\#=<\mathrm{H}, \mathrm{B} \#<==> \\&\quad\mathrm{M}\#=\mathrm{H}, \mathrm{C}\#=\mathrm{B}+\text{CC}, \text{min}(\mathrm{T}, \mathrm{M}, \text{CC}). \end{aligned} $$

The translation of this predicate into words is roughly as follows, first ignoring the counts: M is smaller than a list with head H and tail T, if it is smaller than the tail T and it is smaller than the head, i.e., M ≤H. Now, we also impose that the value M is reached C times as follows: it is reached CC times in the tail and C = B + CC where B is a variable equal to 1 iff M is equal to the head and 0 otherwise. Note that this latest statement is enforced through a reified constraint, it will therefore not lead to immediate branching but to the propagation of as much information as possible (e.g., if some values in the list are already known to be strictly greater than others, the corresponding boolean for each of them will be set to 0, and thus the sum will by necessity enforce some other values to be equal to the required minimum).

This concise and portable implementation will probably improve when the minimum and min_n_ global constraints are available (see [27] for a reference). However it already proves very efficient as demonstrated in the next section.

When C is equal to one, we can fall back to using the built-in min construct in a constraint (e.g., M #= min(L1, min(L2, L3))). Some preliminary benchmarking showed that the reified version is more efficient if the length of the list is greater than 3 or 4.

### Enumeration strategy

Constraints over finite domains come with domain filtering algorithms which dynamically prune the domain of variables when the domain of other variables change in a constraint. However this strategy is not complete and must be combined with a search procedure for virtually enumerating all possible values of the variables. For this application we obtained good performances with dichotomic search by bissecting the domain of the variables (bisect option in SWI-Prolog) without any particular heuristics for choosing the variables.

Note that since this approach is numerical, contrary to solving *symbolically* an exponential but finite number of linear systems as done in Section “A simple example, the Michaelis-Menten reduction” and in [13], there can be an infinite number of solutions. This situation denotes an under-constrained linear system and remains to be interpreted biologically. In practice bounds are put on variables in order to force finiteness. This is not a restriction in practice since biochemical species’ concentrations usually do not vary by more than a hundred of magnitude orders.

Furthermore, in order to speed-up the computation of all solutions in such large domains, we used an iterative domain expansion strategy: the problem is first tried with a domain of [−2,2] for all variables, i.e., equilibrations are searched by rescaling in the 10^−2^,10^2^ interval. If that fails, the domain is doubled and the problem tried again until a limit of 10^−128^,10^128^.

## Computation results on Biomodels.net

To benchmark our approach, we applied it systematically to all the dynamical models of the curated part of the http://biomodels.net repository [16] of biological systems, with *ε* set arbitrarily to 0.1.

We used release *r24* from 2012-12-12 which includes 436 curated models. Among them, only 55 models have non-trivial purely polynomial kinetics (ignoring *events* if any). Our computational results on those are summarized in Table 1, where the first column indicates whether a complete equilibration was found, and the times are in seconds.

**Table 1:** **Number of models of the BioModels repository, with a polynomial kinetics, for which tropical equilibrations were found or not, with corresponding size of the model and computation time**

**Found**	**# models**	**Variables (avg/min/max)**	**Time in seconds**
			**(avg/min/max)**
yes	23	17.348/3/ 86	0.486/0.004/2.803
no	32	17.812/1/194	0.099/0.000/1.934

The domain expansion strategy coupled with dichotomic search by domain bisections allowed us to gain two orders of magnitude of computation time on the biggest models.

Only one of the models (number 002) used values far from 0 in the equilibration (up to *ε*^40^) and has no complete equilibration if the domain is restricted to [−32,32]. This is because the model is written with units such that the initial concentrations are of the order 10^−21^, translating the search accordingly. We thus do not believe that enlarging the domains even more would lead to more equilibrations. Nevertheless, choosing a smaller *ε* might increase the number of equilibrations.

18 of the 23 models for which there is a complete equilibration are actually under-constrained and appear to have an infinity of such solutions (typically linear relations between variables). For the 5 remaining ones, we computed all complete equilibrations as shown in Table 2.

**Table 2:** **Number of equilibrations and computation time for the models of the BioModels database with finitely many numerical solutions**

**Model**	**# variables**	**# equilibrations**	**Total time (s)**
BIOMD0000000002	13	18	53
BIOMD0000000122	14	9	4.1
BIOMD0000000156	3	1	<1ms
BIOMD0000000229	7	1	0.07
BIOMD0000000413	5	5	0.4

## Conclusions

In this paper we have shown that constraint-based methods can be efficiently used to numerically solve tropical equilibration problems in biological models of real-size in the BioModels.net repository. These calulations are important for model reduction and for determining the unknown orders of the variables. Once the orders of the variables are known, the rapid variables can be identified and the system reduced to a simpler one. This truncation, described in Section “Tropical equilibrations and model reductions” coupled with the proposed constraint-based method for finding equilibrations therefore provides an automatic way to reduce models and to identify fast/slow variables. We have started the application of such technique on non-trivial models provided by biologists and modellers and hope to be able to improve both the understanding, through that identification of fast/slow variables, and the analysis, through the size reduction, of those models.

Even with the progress of high-throughput technologies, having more focused models, with fewer species and parameters to measure, will definitely permit an improvement in the quality and speed of development of the models. Furthermore, the structural methods for comparing models in model repositories, such as [7], can be refined by filtering the structural reduction relationships according to the kinetics of the reactions and the tropical reasoning on the magnitude orders.

In many cases, it makes sense biologically to only look for partial equilibrations. Strategies to decide when such decision has to be made remain unclear. Nevertheless the framework of partial constraint satisfaction and more specifically Max-CSP [28] would allow us to easily handle the maximization of the number of equilibrated variables.

One of the limits of this approach, is that it is not particularly well suited to equilibration problems with an infinite number of solutions. As discussed at the end of previous section, in such situations symbolic solutions would be more appropriate. Nevertheless, even the approximate detection of such a case by the very high number of (bounded) numerical solutions was shown to be not very costly in practice.
